# ONX-0914 Suppresses Hormone-Sensitive Prostate Cancer by Promoting O-GlcNAcylation-Mediated Stabilization of TCF7L1

**DOI:** 10.32604/or.2026.073156

**Published:** 2026-03-23

**Authors:** Peng Xian, Zhenwei Feng, Haitao Yu, Hubin Yin, Haonan Chen, Tenglin Shi, Xilai Li, Chunlin Zhang, Xuesong Bai, Xin Gou, Xinyuan Li, Jie Li

**Affiliations:** 1 Department of Urology, The First Affiliated Hospital of Chongqing Medical University, Chongqing, China; 2 Department of Urological Oncology, Chongqing University Cancer Hospital, Chongqing, China; 3 Chongqing Key Laboratory of Molecular Oncology and Epigenetics, Chongqing, China

**Keywords:** Prostate cancer, transcription factor 7–like 1 (TCF7L1), androgen receptor, O-GlcNAcylation, hexosamine biosynthetic pathway

## Abstract

**Objective:**

Androgen receptor (AR) signaling is a central driver of prostate cancer progression, yet the metabolic and transcriptional mechanisms regulating AR expression remain incompletely characterized. This study investigated whether the immunoproteasome inhibitor ONX-0914 suppresses hormone-sensitive prostate cancer (HSPC) through metabolic modulation of AR and aimed to identify the transcriptional mediator involved.

**Methods:**

HSPC and castration-resistant prostate cancer models were used to evaluate the effects of ONX-0914 on cell proliferation, invasion, migration, and epithelial–mesenchymal transition. Xenograft assays, bioinformatic screening, and analyses of O-GlcNAcylation and protein stability were performed, together with quantitative polymerase chain reaction (qPCR) and Western blotting.

**Results:**

ONX-0914 markedly suppressed hormone-sensitive prostate cancer (HSPC) progression through both LMP7-dependent and LMP7-independent mechanisms. Mechanistically, ONX-0914 activated the hexosamine biosynthetic pathway and enhanced global O-GlcNAcylation, leading to stabilization of the transcriptional repressor Transcription factor 7–like 1 (TCF7L1) and consequent suppression of androgen receptor (AR) expression. Functionally, activation of the O-GlcNAcylation–TCF7L1 axis inhibited cell proliferation, invasion, migration, and epithelial–mesenchymal transition *in vitro*. *In vivo*, TCF7L1 overexpression, particularly under conditions of enhanced O-GlcNAcylation, significantly suppressed tumor growth and AR expression.

**Conclusion:**

This study identifies a novel ONX-0914/HBP/TCF7L1 O-GlcNAcylation axis that metabolically stabilizes TCF7L1, leading to repression of AR signaling and inhibition of HSPC progression. These findings reveal a previously unrecognized metabolic–transcriptional regulatory mechanism and highlight TCF7L1 O-GlcNAcylation as a potential therapeutic target in AR-dependent prostate cancer.

## 1 Introduction

Prostate cancer ranks as the second most common malignancy among men worldwide, and represents a major public health burden in male healthcare [1]. Although patients with locally confined prostate cancer generally have a favorable prognosis, the 5-year survival rate for those with metastatic disease declines significantly to approximately 34% [2]. In the stage of metastatic hormone-sensitive prostate cancer (HSPC), the combination of androgen deprivation therapy (ADT) with anti-androgen agents demonstrated strong therapeutic efficacy. In particular, the recent introduction of novel hormonal agents such as enzalutamide and apalutamide has significantly prolonged patient survival [3]. In contrast, metastatic castration-resistant prostate cancer (CRPC) remains difficult to treat, despite the availability of emerging therapies, including poly(ADP-ribose) polymerase inhibitors, androgen degraders, and isotope-based therapies [4]. Androgen receptor (AR) protein is expressed in nearly all primary and metastatic tumors, and its transcriptional activity continues to drive tumor growth even in CRPC [5]. Consequently, dysregulated AR signaling is a central feature of prostate cancer pathogenesis and remains the primary therapeutic target at both the HSPC and CRPC stages.

The immunoproteasome is an inducible proteasomal variant of the proteasome that is predominantly enriched in immune cells, and contains distinct catalytic subunits, such as low-molecular mass polypeptide 7 (LMP7), which play essential roles in MHC class I antigen presentation [6]. Within the tumor microenvironment, immunoproteasomes may contribute to antitumor immune responses by enhancing the presentation of tumor antigens [7]. As a selective immunoproteasome inhibitor, ONX-0914 is thought to exert antitumor effects by modulating immune cell function in the tumor microenvironment; however, its precise mechanisms of action remain incompletely understood [8,9]. Notably, several studies have reported that ONX-0914 can elicit anticancer effects not solely dependent on immunoproteasome inhibition [10]. Our previous work demonstrated that ONX-0914 significantly reduced tumor-elicited inflammatory responses and suppressed CRPC progression in tumor graft models through its inhibition of LMP7 [11]. Similarly, treatment with ONX-0914 in a prostate cancer–specific mouse model led to a significant reduction in prostate tumor growth, a decreased incidence of malignant lesions, and complete suppression of metastasis [12]. Despite these findings, it remains unclear whether ONX-0914 exerts comparable biological effects in HSPC and whether distinct molecular mechanisms account for its efficacy in HSPC versus CRPC.

O-GlcNAcylation is widely recognized as a metabolically responsive and dynamic type of post-translational modification that links cellular metabolism to signal transduction. Through the hexosamine biosynthetic pathway (HBP), glucose, glutamine, and other nutrients are converted into uridine diphosphate N-acetylglucosamine (UDP-GlcNAc), which serves as the substrate for the addition of O-linked β-N-acetylglucosamine [13]. This modification of serine and threonine residues on nuclear and cytoplasmic proteins is catalyzed by O-GlcNAc transferase (OGT), whereas the removal of O-GlcNAc is mediated by the glycosidase O-GlcNAcase (OGA) [14]. Glutamine: fructose-6-phosphate amidotransferase 1 (GFAT1) is a key rate-limiting enzyme that regulates metabolic flux through the HBP and plays a pivotal role in controlling this pathway [15]. Reduced GFAT1 expression has been associated with poor prognostic outcomes in several cancers [16]. O-GlcNAcylation functions as a metabolic sensor that modulates key signal transduction pathways and transcription factors. Although O-GlcNAcylation has been extensively studied in cancer and is commonly related to tumor-promoting effects [17], its biological role is not unidirectional. Emerging evidence indicates that O-GlcNAcylation can also exert tumor-suppressive effects, depending on the cellular context, tumor type, and specific molecular targets [18].

Transcription factor 7–like 1 (TCF7L1) is a member of the T-cell factor/lymphoid enhancer factor (TCF/LEF) family of Wnt-responsive transcription factors [19]. It plays critical roles in regulating stem cell pluripotency and lineage commitment [20,21]. TCF7L1 is mainly characterized as a transcriptional repressor of Wnt target genes [22]. Aberrant activation of the Wnt signaling pathway has been implicated in the initiation and progression of a wide range of malignancies [23,24]. Particularly, within prostate cancer contexts, Wnt signaling pathway activation exhibits a link to the enhancement of AR signal [25]. Notably, TCF7L1 exhibits a bimodal function, acting as either a tumor promoter or suppressor depending on the cancer type [26,27]. This functional diversity is closely associated with its expression levels in different tumor microenvironments, post-translational modifications and interacting protein networks.

The objective of this study was to determine whether ONX-0914 modulates HBP activity and O-GlcNAcylation, stabilizes TCF7L1, and suppresses AR signaling in HSPC. We further aimed to investigate whether these effects occur beyond classical LMP7 dependence, thereby revealing a distinct metabolic–epigenetic mechanism beyond classical immunoproteasome inhibition.

## 2 Materials and Methods

All key reagents, antibodies, plasmids, cell lines, and chemicals used in this study are summarized in Supplementary Tables S1–S9, including supplier information and catalog numbers. Detailed experimental procedures are described in the subsequent sections.

### 2.1 Animals

A total of 70 six-week-old male BALB/c nude mice (18–20 g) were procured from the AAALAC-accredited MODEL ORGANISMS facility (Shanghai, China) and maintained in specific pathogen-free (SPF) environments, with ad libitum access to standard chow and sterile water. All animal procedures were approved by the Institutional Animal Care and Use Committee (IACUC) of Chongqing Medical University (Approval No: 2020-754), and conducted in accordance with the Animal Research: Reporting of *In Vivo* Experiments (ARRIVE) guidelines. Mice were randomly assigned to experimental groups (*n* = 5 per group) using a computer-generated random number sequence (simple randomization). Investigators responsible for tumor measurement and data analysis were blinded to group allocation using coded identifiers until completion of the primary analysis. PC3 or LNCaP cells in the logarithmic growth phase were harvested, counted, and resuspended in sterile culture medium at a density of 5 × 10^6^ cells per 100 µL. The cell suspensions were injected subcutaneously into the right flank of each mouse using a 1-mL, 27-gauge sterile syringe with slow administration. Mice were individually monitored for 30 min post-injection to ensure the absence of acute adverse reactions. In the LNCaP xenograft experiment, 10 of 30 inoculated mice failed to develop measurable tumors and were excluded prior to treatment. When tumor volumes reached approximately 100 mm^3^, mice were treated with ONX-0914 (Selleck Chemicals, S7172, Houston, TX, USA) administered intraperitoneally at a dose of 3 mg/kg, twice weekly, for 3 weeks. Mice in the control group received intraperitoneal injections of DMSO according to the same schedule. Although a formal power analysis was not conducted, the sample size and dosing regimen were determined based on previously published ONX-0914 xenograft studies and commonly accepted practices for mechanistic *in vivo* experiments [11]. Under these conditions, statistically significant differences in tumor growth were observed between groups. Tumor growth was periodically evaluated at twice-weekly intervals by measuring tumor length (L) and width (W) via digital calipers. Tumor volume (V) was calculated using the established formula: V = 0.52 × L × W^2^. Predefined humane endpoints were established, including tumor volume exceeding 1500 mm^3^, tumor ulceration, or body weight loss greater than 15%. At the experimental endpoints, mice were euthanized via pentobarbital administration (Sigma-Aldrich, P3761, St. Louis, USA) and tumor tissues were collected for downstream analyses and imaging (Supplementary Table S1).

### 2.2 Cell Culture

PC3, LNCaP, and HEK293T cells were obtained from the Cell Bank of the Chinese Academy of Sciences (Shanghai, China), authenticated by short tandem repeat (STR) profiling, and confirmed to be mycoplasma-free. Cells were cultured in RPMI-1640 medium (Gibco, 11875-093, Thermo Fisher Scientific, Waltham, MA, USA) supplemented with 10% fetal bovine serum (FBS, Procell, 164210, Wuhan, China), 1 mmol/L glutamine, and 100 µg/mL streptomycin plus 100 U/mL penicillin (both antibiotics: Beyotime, C0222, Shanghai, China). All cell lines were incubated at 37°C in a humidified 5% CO_2_ environment. For assessing immunoproteasome inhibition in prostate cancer cells, PC3 and LNCaP cells were treated with DMSO or ONX-0914 (300 nM) for 24 h. The selected concentration and treatment duration were based on previously published studies in prostate cancer demonstrating effective biological activity of ONX-0914, and were further supported by our preliminary optimization experiments [11] (Supplementary Table S2).

### 2.3 Plasmids, Cell Transfection, and Lentiviral Infection

The ubiquitin (Ub) gene was cloned into the pcDNA3.1-Flag vector (Invitrogen, Waltham, USA). The full-length human TCF7L1 coding sequence was subcloned into the PLVX-EF1α-Puro vector (Clontech, Mountain View, CA, USA) and verified by Sanger sequencing. LMP7 knockdown was achieved using three independent shRNAs cloned into the PLVX-Puro vector (Clontech, Mountain View, CA, USA), with target sequences listed in Supplementary Table S3. The knockdown efficiency of each shRNA was evaluated, and the most efficient shRNA was selected for subsequent experiments.

Plasmid and shRNA transfections were performed using Lipofectamine 3000 (Invitrogen, L3000015, Thermo Fisher Scientific, Waltham, MA, USA) following the manufacturer’s instructions. Cells were transfected at 60%–70% confluency using 2.5 μg of plasmid DNA per well of a 6-well plate. Lentiviral particles were produced in HEK293T cells by co-transfection with the packaging plasmids psPAX2 and pMD2.G (Addgene, Watertown, MA, USA). PC3 and LNCaP cells were infected with viral supernatants in the presence of 8 μg/mL polybrene (Sigma-Aldrich, H9268, St. Louis, MO, USA) at an MOI of 5 for 24 h and subsequently selected with puromycin (2 μg/mL; Hanbio, HB-PU-500, Shanghai, China) to generate stable cell lines. Knockdown and overexpression efficiencies were confirmed by Western blotting and quantitative polymerase chain reaction (qPCR). A non-targeting (scrambled) shRNA lentivirus was used as the knockdown control, and the corresponding empty vector was used as the control for overexpression experiments (Supplementary Table S4).

### 2.4 Real-Time Quantitative PCR

Total RNA was extracted from tissues and cells using TRIzol reagent (ABclonal, RK30129, Wuhan, China). RNA concentration and purity were assessed using a NanoDrop spectrophotometer by measuring A260/280 ratios prior to reverse transcription. cDNA was synthesized by reverse transcription using ABScript Neo RT Master Mix for qPCR which includes a genomic DNA remover (ABclonal, RK20433, Wuhan, China). Real-time qPCR was performed on a QuantStudio 6 Flex Real-Time PCR System (Applied Biosystems) using SYBR Green Fast qPCR Mix (ABclonal, RK21203, Wuhan, China) in a total reaction volume of 20 μL (Supplementary Table S5). The thermal cycling conditions were as follows: initial denaturation at 95°C for 30 s, followed by 40 cycles of 95°C for 5 s and 60°C for 30 s. Gene expression levels were calculated using the 2^−ΔΔCt^ method and normalized to β-actin. qPCR analyses were performed using three independent biological replicates, with each sample analyzed in technical triplicates. Primer sequences are provided in Supplementary Table S3.

### 2.5 Western Blotting

Mouse tumor grafts and ONX-0914–treated PC3/LNCaP cells were lysed in radioimmunoprecipitation assay (RIPA) buffer supplemented with a protease inhibitor cocktail. Thirty micrograms of total protein per sample were separated by 10% SDS-PAGE on polyacrylamide gels, and transferred onto PVDF membranes. After blocking with 5% nonfat milk at room temperature for 1 h, membranes were incubated with primary antibodies overnight at 4°C, followed by incubation with the corresponding secondary antibodies (1:3000, Boster Biological Technology, BA1050, BA1054, Pleasanton, CA, USA) at room temperature for 1 h on the following day. Protein signals were visualized using Fusion FX6 Chemiluminescence Imaging System (Vilber Lourmat Deutschland GmbH, Eberhardzell, Germany), and expression levels were normalized to β-actin. Antibodies used in this study included GFAT1 (1:1000, 14132-1-AP), AR (1:1000, 22089-1-AP), TCF7L1 (1:1000, 14519-1-AP), Flag (1:1000, 20543-1-AP), anti-HA (hemagglutinin) tag antibody (1:1000, 51064-2-AP), E-cadherin (1:1000, 20874-1-AP), N-cadherin (1:1000, 22018-1-AP), vimentin (1:1000, 10366-1-AP), and β-actin (1:30,000, 66009-1-Ig) from Proteintech Group (Chicago, IL, USA), as well as O-GlcNAc antibody (1:1000, Jingjie PTM BioLab, PTM-952, Hangzhou, China) (Supplementary Table S6).

### 2.6 SWGA Affinity Pull-Down and Protein Stability Assay

Cell lysates were denatured in glycoprotein denaturation buffer at 100°C for 10 min and treated with PNGase F (500 U per reaction, New England Biolabs, P0704S, Ipswich, MA, USA) at 37°C for 1 h to remove N-linked glycosylation. The treated samples were incubated overnight at 4°C with biotinylated sWGA magnetic beads (Vector Laboratories, B-1025S-5, Newark, CA, USA). To evaluate pull-down specificity, a bead-only control (magnetic beads without sWGA) was included, and a competitive sugar control was performed by pre-incubating lysates with free GlcNAc (0.2 M) for 30 min at 4°C prior to sWGA pull-down. Bead-bound proteins were eluted by heating at 95°C for 10 min in 2× SDS loading buffer. O-GlcNAc modification levels of TCF7L1 were assessed by Western blotting, with total TCF7L1 protein serving as a loading control. For protein stability analysis, LNCaP cells were transfected with TCF7L1-HA. Following treatment with cycloheximide (CHX; Sigma-Aldrich, C7698, St. Louis, MO, USA) at 50 ug/mL for 0, 6, 12, and 24 h, proteins were extracted and analyzed by Western blotting using an anti-HA antibody (Supplementary Table S7).

### 2.7 Co-Immunoprecipitation Assay

LNCaP cells were co-transfected with TCF7L1-HA and Ub-Flag. After transfection, cells were treated with OSMI-1 (50 μM, Selleck Chemicals, S9835, Houston, USA) or PUGNAc (100 μM, Sigma-Aldrich, A7229, St. Louis, USA) for 24 h. Cells were lysed on ice using ice-cold IP lysis buffer (Beyotime Biotechnology, P0013, Shanghai, China) supplemented with a protease inhibitor cocktail and freshly added deubiquitinase inhibitor N-ethylmaleimide (Sigma-Aldrich, E3876, St. Louis, USA). Lysates were clarified via centrifugation at 12,000× *g* for 15 min at 4°C, and supernatants were collected. An aliquot of each supernatant was reserved as input. Supernatants were incubated overnight at 4°C with 2.5 μg of anti-HA antibody (or equivalent control IgG) under gentle rotation. Subsequently, 30 μL of Protein A/G magnetic beads (Thermo Fisher Scientific, 88803, Waltham, MA, USA) were added, and incubation continued for an additional 8 h at 4°C. The extended incubation time with Protein A/G beads was optimized to maximize immunoprecipitation efficiency while minimizing nonspecific binding. Beads were washed four times with IP lysis buffer, and immune complexes were eluted by boiling in SDS sample buffer. Eluted proteins were separated by SDS-PAGE and analyzed by immunoblotting with an anti-Flag antibody to detect ubiquitinated TCF7L1 (Supplementary Table S8).

### 2.8 Cell Viability Assay (CCK-8)

Cell viability was assessed using the Cell Counting Kit-8 (CCK-8; Dojindo Laboratories, CK04, Kumamoto, Japan) according to the manufacturer’s instructions. Briefly, cells were seeded into 96-well plates at a density of 2 × 10^3^ cells per well in 100 μL serum-free medium (Gibco, 11875-093, Thermo Fisher Scientific, Waltham, MA, USA) and allowed to attach for 12 h. The medium was then replaced with serum-free medium containing the indicated treatments, and cells were incubated for 24 h. After treatment, the medium was aspirated and replaced 90 μL of serum-free medium and 10 μL of CCK-8 reagent were added, followed by incubation at 37°C for 1 h. Blank wells containing medium and CCK-8 reagent without cells were included for background subtraction. Absorbance at 450 nm was measured using a microplate reader (Bio-Rad Laboratories, Hercules, CA, USA). All conditions were tested in triplicate, and each experiment was repeated at least three times (Supplementary Table S9).

### 2.9 Colony Formation Assay

Cells were seeded into 6-well plates at a density of 2000 cells per well in 2 mL complete medium and allowed to attach for 12 h. The medium was then replaced with fresh complete medium containing the indicated treatments and cells were incubated for 24 h. Subsequently, the medium was changed to drug-free complete medium and refreshed every 2 days. After a total incubation period of 14 days, the medium was aspirated and colonies were gently washed twice with PBS, fixed with 4% paraformaldehyde at room temperature for 15 min, and stained with 0.1% crystal violet for 15 min. Plates were rinsed thoroughly with distilled water to remove excess stain and air-dried. Colonies were photographed, and colonies containing ≥50 cells were counted using ImageJ (version 1.54 f, National Institutes of Health). All conditions were tested in triplicate, and each experiment was repeated at least three times (Supplementary Table S9).

### 2.10 Migration and Invasion Assays

Migration assays were performed using Transwell inserts with an 8 μm pore size (Corning Incorporated, 3422, Corning, NY, USA). For migration, 1 × 10^5^ cells were resuspended in 200 μL of serum-free medium and added to the upper chamber of each insert, while the lower chamber contained 700 μL of medium supplemented with 10% FBS as a chemoattractant. Plates were incubated at 37°C in a humidified 5% CO_2_ atmosphere for 24 h. Migration and invasion assays were performed as independent experiments using identical cell numbers and incubation times, except for the Matrigel coating applied in invasion assays. For invasion assays, Transwell inserts were precoated with Matrigel (Corning, 356234, NY, USA) diluted 1:8 in serum-free medium (50 μL per insert) and allowed to polymerize at 37°C for 1 h prior to cell seeding. After incubation, non-migrated or non-invaded cells on the upper membrane surface were removed with a cotton swab. Migrated or invaded cells on the lower surface were fixed in 4% paraformaldehyde for 15 min, and stained with 0.2% crystal violet for 10–20 min. Stained membranes were imaged under a light microscope at 200× magnification. Representative images were captured, and quantitative analysis was performed using ImageJ software (version 1.54f, National Institutes of Health), counting cells in five random fields per membrane and calculating the average (Supplementary Table S9).

### 2.11 Venn Diagram and Intersection Analysis

AR-related transcription factors (TFs) were obtained from the JASPAR database (JASPAR 2022, CORE collection; https://jaspar.genereg.net/). TFs were defined as AR-related if they were annotated in JASPAR as having predicted binding motifs associated with androgen receptor–mediated transcriptional regulation. Differentially expressed genes (DEGs) were identified from the TCGA-PRAD cohort and the GSE46602 dataset. For the TCGA-PRAD cohort, raw count data were normalized and analyzed using the DESeq2 package in R, which applies a negative binomial generalized linear model for differential expression analysis. For the GSE46602 microarray dataset, background correction and normalization were performed using the limma package, followed by linear modeling and empirical Bayes moderation. Genes with a false discovery rate (FDR) < 0.05 and an absolute log_2_ fold change (|log_2_FC|) > 1 were considered significantly differentially expressed. Intersection analysis was performed to identify overlapping genes among JASPAR-derived AR-related TFs and DEGs from both TCGA-PRAD and GSE46602 datasets. Venn diagrams were generated in R (version 4.2.2) using the VennDiagram package to visualize the shared transcription factors.

### 2.12 Statistical Analysis

All *in vitro* experiments were conducted with a minimum of three independent biological replicates. Statistical analyses were performed using GraphPad Prism 9 (GraphPad Software, San Diego, CA, USA). Data are presented as mean ± standard deviation (SD) from at least three independent experiments. Normality of data distribution was assessed using the Shapiro–Wilk test prior to parametric testing. For comparisons between two groups, an unpaired two-tailed Student’s *t*-test was used. For comparisons involving three or more groups, one-way analysis of variance (ANOVA) was first conducted, followed by Tukey’s multiple comparison test for post-hoc analysis where appropriate. Statistical significance was defined as a *p*-value < 0.05.

## 3 Results

### 3.1 ONX-0914 Suppresses Proliferation, Migration, and Epithelial–Mesenchymal Transition in HSPC through Mechanisms That Are Not Solely Dependent on LMP7

Given the context-dependent effects of ONX-0914, we first examined basal LMP7 expression in prostate cancer models. Quantitative PCR and Western blot analyses revealed that LMP7 expression was significantly higher in PC3 cells than in LNCaP cells (Supplementary Fig. S1A,B). LMP7 knockdown was achieved using three independent shRNAs and confirmed by qPCR and Western blotting in both LNCaP and PC3 cells, and sh1 was selected for all subsequent experiments (Supplementary Fig. S1C,D). Notably, ONX-0914 did not significantly affect LMP7 expression in cultured cells, whereas shLMP7 robustly reduced LMP7 levels with or without ONX-0914 co-treatment (Supplementary Fig. S1E,F).

**Figure 1:**
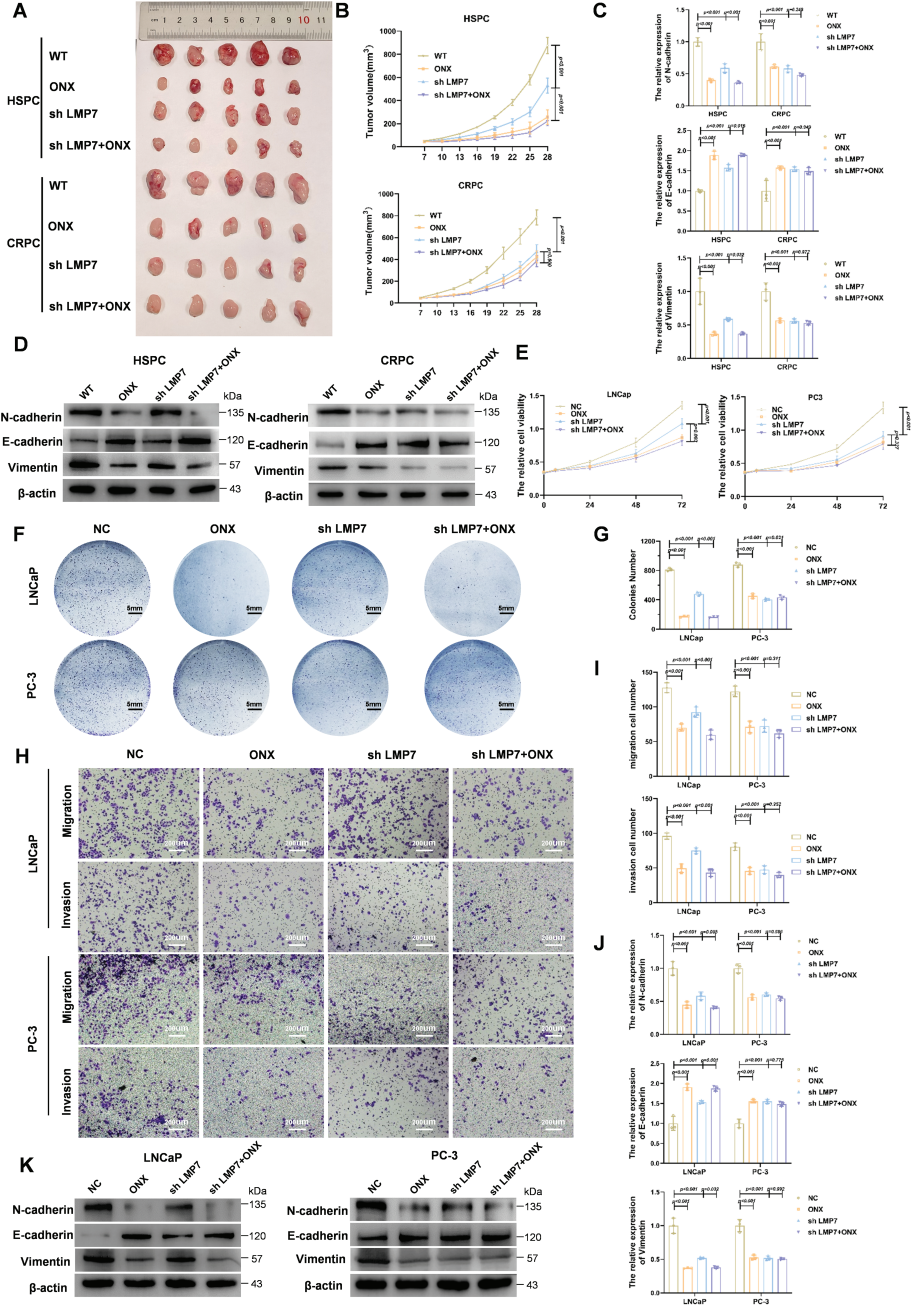
ONX-0914 suppresses the proliferation, migration, and EMT through mechanisms that are not solely dependent on LMP7 in HSPC. (**A**) Tumor images from xenograft mice injected with LNCaP or PC3 cells and treated with indicated conditions. (**B**) Tumor volume growth curves over time in each treatment group. (**C**) qPCR analysis of EMT marker expression in LNCaP- and PC3-derived tumors. (**D**) Immunoblot profiling of EMT-related proteins in xenograft samples. (E) Cell metabolic activity assessed using the CCK-8 assay. (**F**) Representative images of cultured LNCaP and PC3 cells under the indicated treatments. (**G**) Statistical analysis of the number of cell colonies in (**F**). (**H**) Transwell micrographs illustrating invasive and migratory behavior of LNCaP and PC3 cells. (**I**) Quantification of transwell migration and invasion assays. (**J**) qPCR analysis of EMT markers in LNCaP and PC3 cells. (**K**) Immunoblot profiling of EMT-associated proteins in cultured LNCaP and PC3 cells.

Based on these observations, we next evaluated the antitumor effects of ONX-0914 in subcutaneous xenograft models generated from hormone-sensitive (LNCaP) and castration-resistant (PC3) prostate cancer cell lines, with or without LMP7 knockdown. Efficient LMP7 knockdown was confirmed by qPCR and Western blot (Supplementary Fig. S1G,H). In the HSPC model, ONX-0914 significantly reduced tumor volume compared with the control or LMP7 knockdown alone, and the combination of ONX-0914 with LMP7 knockdown produced further inhibition (Fig. 1A,B). In contrast, in the CRPC model, ONX-0914 and LMP7 knockdown exhibited similar growth suppression, with no additive effect observed.

To evaluate epithelial–mesenchymal transition (EMT) in tumors, qPCR and Western blot analyses were performed for key EMT markers. In CRPC tumors, the combination of LMP7 knockdown and ONX-0914 did not further inhibit EMT beyond the effect of ONX-0914 alone (Fig. 1C,D). In contrast, in HSPC tumors, the combination treatment further suppressed EMT compared with LMP7 knockdown alone (Fig. 1C,D). These results suggest that ONX-0914 inhibits CRPC predominantly through LMP7 targeting, while in HSPC, ONX-0914 appears to engage additional antitumor mechanisms beyond LMP7 targeting.

To further substantiate the differential dependence on LMP7, *in vitro* experiments were performed using PC3 and LNCaP cells. CCK-8 assays showed that in PC3 cells, LMP7 knockdown, ONX-0914 treatment, and their combination all reduced proliferation relative to the negative control (NC), with no significant differences among the treatments (Fig. 1E), consistent with the results of the colony formation assay (Fig. 1F,G). In contrast, in LNCaP cells, ONX-0914 and the combination group significantly inhibited proliferation compared with the shLMP7 group (Fig. 1E–G).

Transwell assays demonstrated that invasion and migration were reduced under all treatments in both PC3 and LNCaP cells (Fig. 1H,I). Notably, in LNCaP cells, ONX-0914 treatment alone or in combination with shLMP7 resulted in greater inhibition than shLMP7 alone.

EMT marker expression *in vitro*, assessed by qPCR (Fig. 1J) and Western blot (Fig. 1K), mirrored the *in vivo* findings. In LNCaP cells, ONX-0914 and the combination elicited stronger downregulation of N-cadherin and vimentin and augmentation of E-cadherin, whereas in PC3 cells, all treatments produced comparable effects.

Collectively, these results show that ONX-0914 inhibits tumor growth in both HSPC and CRPC models, with a relatively stronger effect in HSPC. Notably, ONX-0914 remains effective in HSPC even under conditions of low LMP7 expression, suggesting that its antitumor activity is not solely dependent on LMP7 inhibition.

### 3.2 ONX-0914 Inhibits Prostate Cancer Progression by Upregulating GFAT1 and Activating the HBP

**Figure 2:**
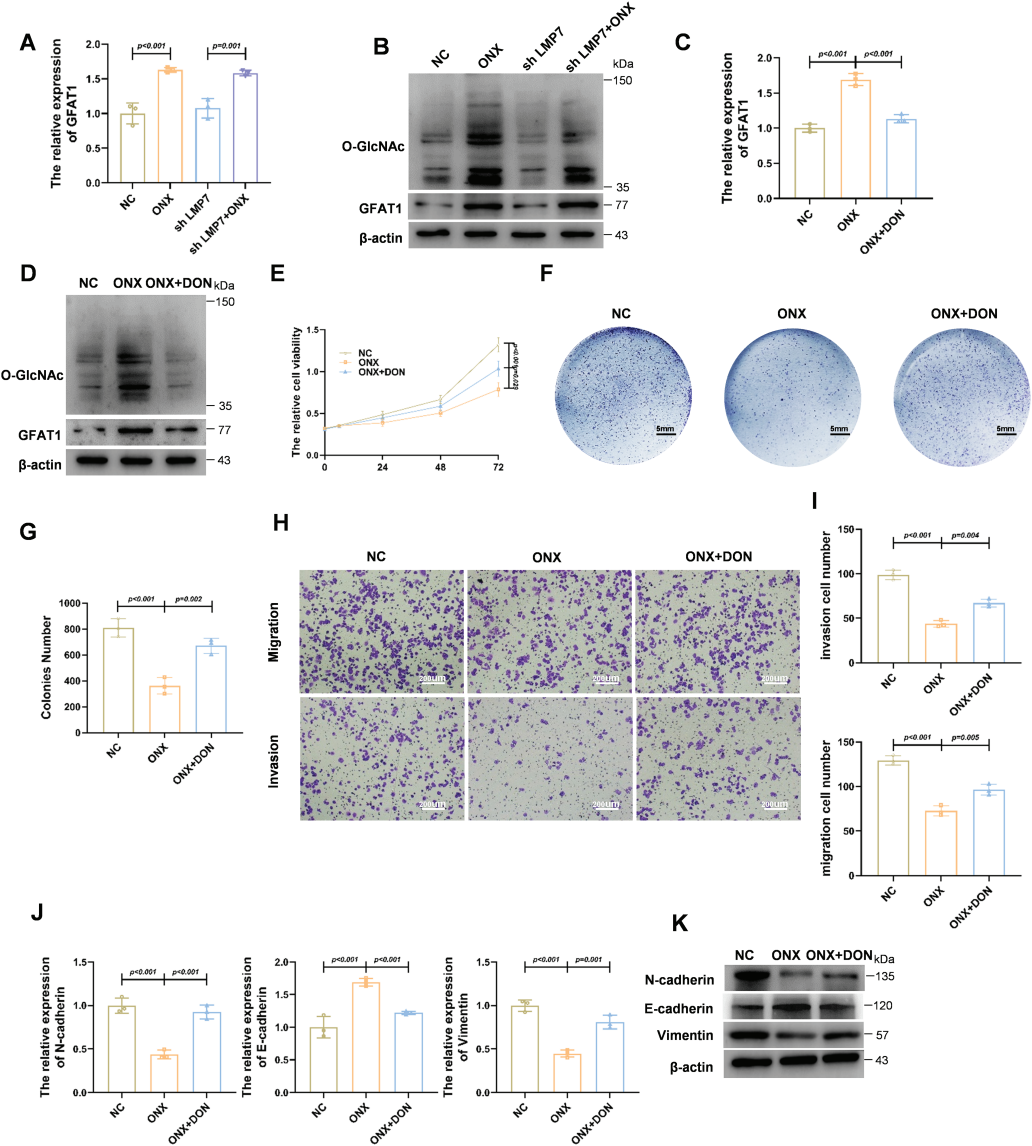
ONX-0914 Inhibits Prostate Cancer Progression by Upregulating GFAT1 to Promote the HBP. (**A**) qPCR analysis of transcripts in LNCaP cells exposed to NC, shLMP7, ONX-0914, or shLMP7 + ONX-0914. (**B**) Immunoblot assessment of O-GlcNAc modification and GFAT1 protein abundance under the same treatments. (**C**) qPCR measurement of GFAT1 mRNA levels in LNCaP cells treated with NC, ONX-0914, or ONX-0914 + DON. (**D**) Immunoblot evaluation of O-GlcNAc and GFAT1 proteins in cells treated as in (**C**). (**E**) Cell metabolic activity determined by the CCK-8 assay in LNCaP cells subjected to the indicated treatments. (**F**) Representative cell culture plate images showing proliferation differences under indicated treatments. (**G**) Quantification of colonies numbers in each treatment group. (**H**) Representative images of transwell invasion and migration assays in LNCaP cells treated with NC, ONX-0914, or ONX-0914 + DON. (**I**) Quantitative analysis of transwell assay results. (**J**) qPCR profiling of EMT-related gene expression in each treatment group. (**K**) Immunoblot detection of EMT-associated proteins in each treatment group.

Considering that ONX-0914 exhibited stronger antitumor effects in HSPC compared with CRPC, we investigated whether metabolic regulation contributes to this differential response. We focused on the HBP, a metabolic route that senses nutrient status, known to regulate O-GlcNAcylation and tumor progression. Treatment of LNCaP cells with ONX-0914 led to a significant increase in GFAT1 levels—the key regulatory enzyme of the HBP controlling pathway flux—demonstrated by both qPCR and Western blot analyses (Fig. 2A,B). Correspondingly, global protein O-GlcNAcylation levels were increased. These effects were more pronounced in cells treated with ONX-0914 or the combination of shLMP7 and ONX-0914 than in shLMP7 alone, indicating that HBP activation is not solely attributable to LMP7 suppression. These biochemical changes are consistent with ONX-0914–mediated activation of the HBP/O-GlcNAc axis in HSPC cells. Although direct measurements of metabolic flux were not performed, HBP activation in this study was inferred from increased GFAT1 expression accompanied by increased global and TCF7L1-specific O-GlcNAcylation. Together, these findings position the HBP/O-GlcNAc pathway as a downstream response associated with ONX-0914 treatment in HSPC cells.

To further assess the functional involvement of GFAT1-mediated HBP activation, cells were co-treated with ONX-0914 and DON (6-Diazo-5-oxo-L-norleucine, a GFAT1 inhibitor), the ONX-0914–induced upregulation of GFAT1 was reversed (Fig. 2C,D). Cell proliferation assays demonstrated that the inhibitory effect of ONX-0914 on HSPC cells was partially attenuated by co-treatment with DON (Fig. 2E–G). Similarly, Transwell assays also showed that ONX-0914 significantly suppressed cell migration and invasion, and these inhibitory effects were partially reversed when combined with DON (Fig. 2H,I). Together, these results suggest that activation of HBP is required for the full antitumor activity of ONX-0914.

Moreover, the inhibitory effects of ONX-0914 on EMT were partially reversed by DON treatment, as shown by both qPCR and Western blot analyses (Fig. 2J,K). Collectively, these findings demonstrate that ONX-0914 exerts its tumor-suppressive effects, at least in part, by upregulating GFAT1 and activating the HBP.

### 3.3 ONX-0914 Downregulates AR Expression and Modulates AR-Dependent Malignant Behaviors in HSPC Cells through HBP Activation

**Figure 3:**
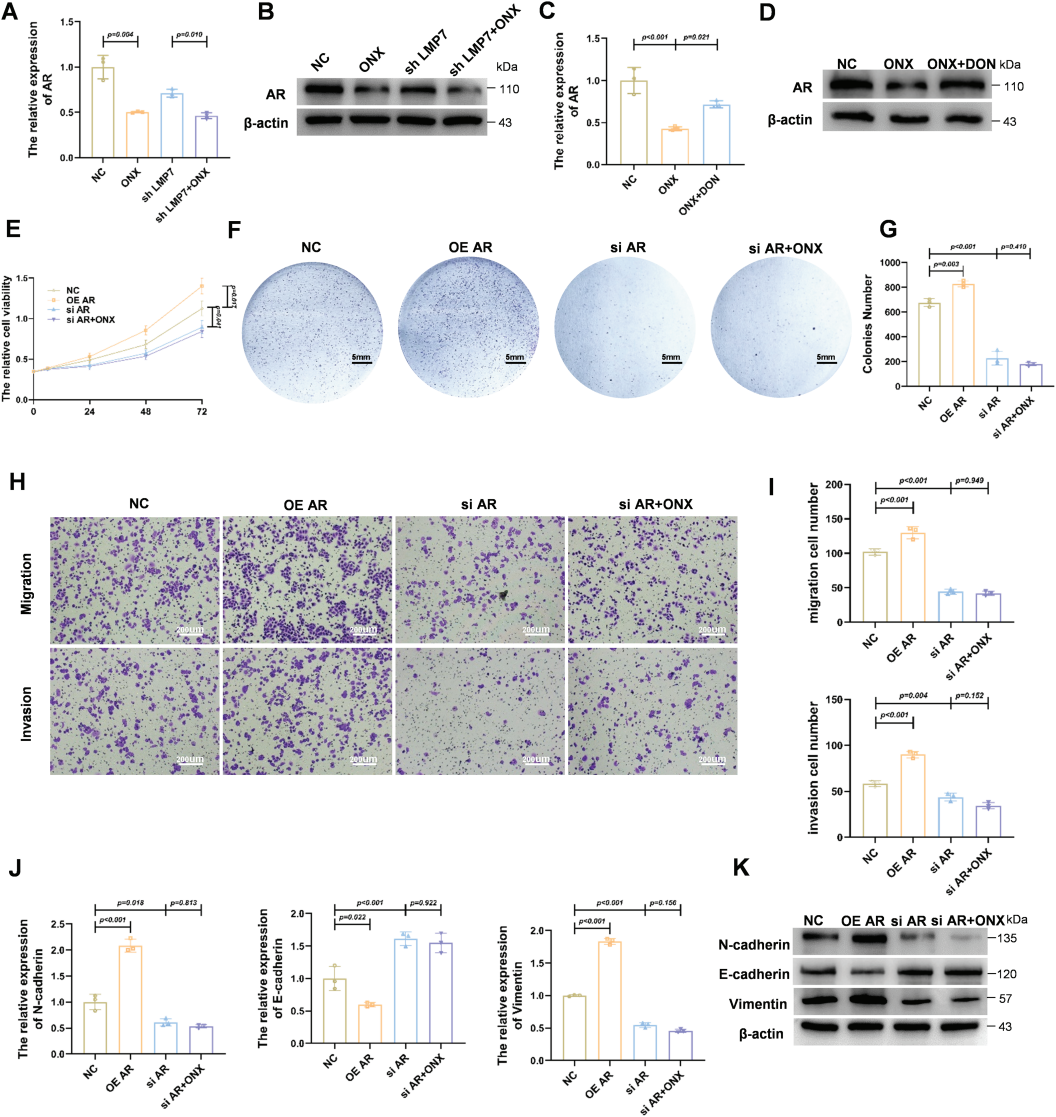
ONX-0914 downregulates AR expression and modulates AR-dependent malignant behaviors in HSPC cells via HBP. (**A**) qPCR quantification of AR transcript levels in LNCaP cells treated with NC, shLMP7, ONX-0914, or shLMP7 + ONX-0914. (**B**) Immunoblot detection of AR protein under the conditions described in (**A**). (**C**) qPCR analysis of AR transcript levels in cells exposed to NC, ONX-0914, or ONX-0914 + DON. (**D**) Immunoblot evaluation of AR protein in the same treatment groups. (**E**) Cell viability analysis by CCK-8 assay of AR overexpression (OE AR), AR knockdown (si AR), and si AR + ONX-0914 on LNCaP cell proliferation. (**F**) Representative cell culture images in the same groups. (**G**) Quantification of cell colonies in each group. (**H**) Representative images of transwell invasion and migration assays in LNCaP cells under AR modulation with or without ONX-0914. (**I**) Quantification of invasion and migration results. (**J**) qPCR profiling of EMT-related gene expression in NC, OE AR, ONX-0914, and si AR + ONX-0914 groups. (**K**) Immunoblot analysis of EMT-associated proteins in the same treatment groups.

Given the central role of AR in driving prostate cancer progression, particularly in HSPC cells, we examined whether ONX-0914 influences AR expression. Western blot analysis in LNCaP cells revealed that AR protein levels were modestly reduced by LMP7 knockdown and further decreased in cells treated with ONX-0914 or the combination of ONX-0914 and shLMP7 (Fig. 3B). These findings were consistent with AR mRNA expression levels measured by qPCR (Fig. 3A), suggesting that ONX-0914 suppresses AR expression more effectively than LMP7 knockdown alone.

To investigate whether AR downregulation is mediated through the HBP, LNCaP cells were treated with ONX-0914 in the presence or absence of DON. As illustrated in Fig. 3C,D, DON partially reversed the ONX-0914–induced suppression of AR expression at both the mRNA and protein levels, indicating that HBP activation contributes to AR downregulation.

We next evaluated the functional role of AR suppression in mediating the antitumor effects of ONX-0914. Overexpression of AR (OE AR) significantly promoted LNCaP cell proliferation, as demonstrated by CCK-8 assays (Fig. 3E) and increased colony formation (Fig. 3F,G) compared with the NC. In contrast, AR knockdown (siAR), either alone or combined with ONX-0914 treatment (siAR + ONX-0914), markedly inhibited cell growth. Notably, the combination of siAR and ONX-0914 did not further suppress proliferation compared with siAR alone, indicating that AR downregulation is a key mechanism underlying ONX-0914–mediated inhibition of cell proliferation.

Similarly, cell invasion and migration assays demonstrated that AR knockdown reduced the invasive and migratory abilities of LNCaP cells, and co-treatment with ONX-0914 did not produce any additional inhibition (Fig. 3H,I).

To further confirm the role of AR in ONX-0914–mediated EMT suppression, we examined EMT marker expression under different AR expression conditions. qPCR analysis (Fig. 3J) showed that AR knockdown reduced the levels of the mesenchymal markers N-cadherin and vimentin while increasing the epithelial marker E-cadherin. Notably, combining AR knockdown with ONX-0914 treatment did not enhance EMT inhibition. These results were corroborated by Western blot analysis (Fig. 3K).

Collectively, these findings demonstrate that ONX-0914 suppresses AR expression via the HBP, and that its inhibitory effects on prostate cancer cell proliferation, invasion, migration, and EMT are mediated through AR downregulation.

### 3.4 ONX-0914 Inhibits Prostate Cancer Progression by Enhancing TCF7L1 O-GlcNAcylation and Suppressing AR Expression

**Figure 4:**
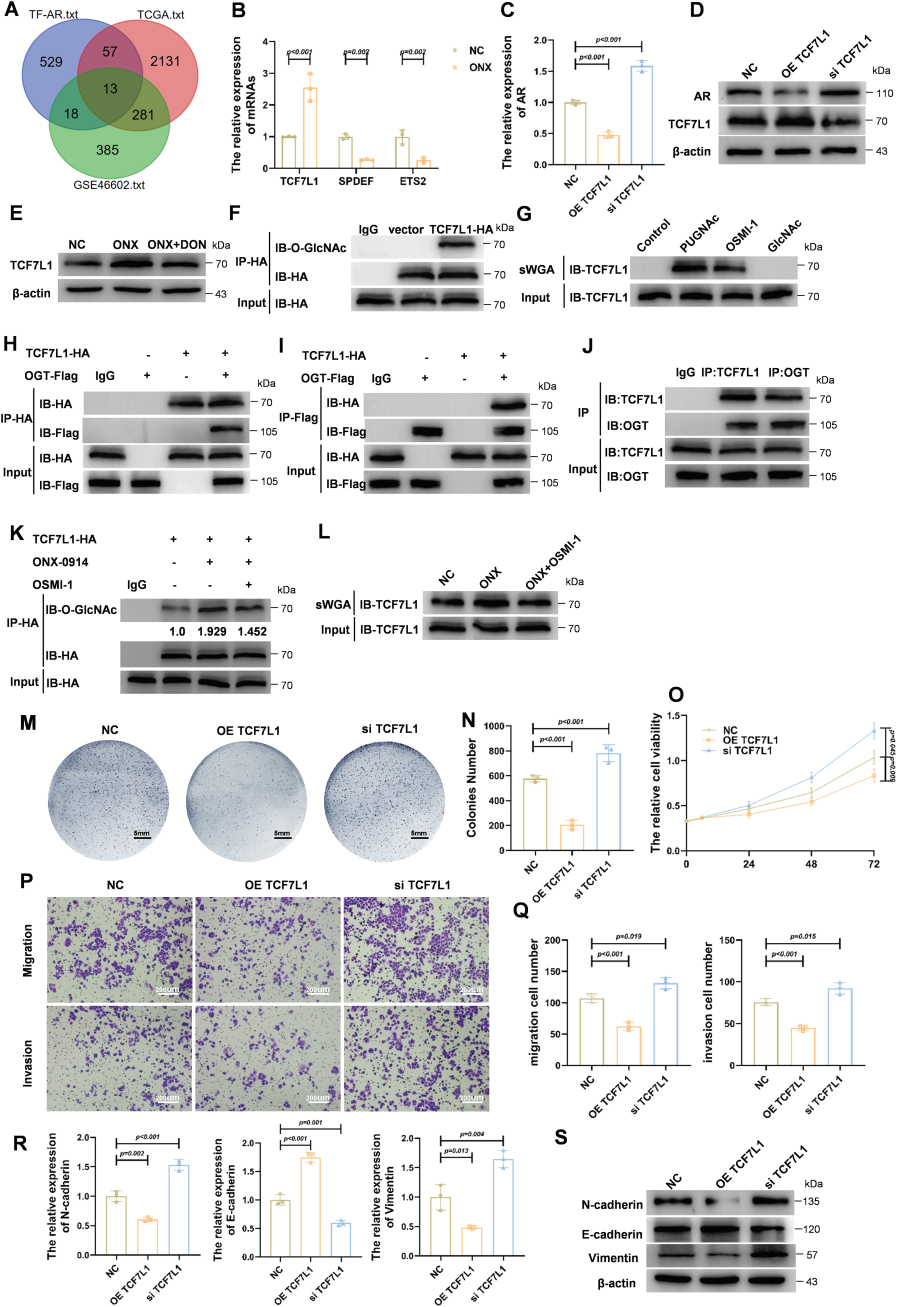
ONX-0914 inhibits prostate cancer progression by promoting TCF7L1 O-GlcNAcylation and subsequently suppressing AR expression. (**A**) Venn diagram showing 13 overlapping AR-associated transcription factors identified from bioinformatic analyses integrating JASPAR, TCGA, and GEO datasets comparing AR-high vs. AR-low prostate cancer samples. (**B**) AR expression changes of candidate transcription factors in LNCaP cells treated with ONX-0914, based on qPCR screening. (**C**) qPCR analyses showing that AR expression is suppressed following TCF7L1 overexpression (OE TCF7L1) and upregulated following TCF7L1 knockdown (si TCF7L1). (**D**) Immunoblot analysis confirming AR expression is suppressed following OE TCF7L1 and upregulated following si TCF7L1. (**E**) Immunoblot analysis showing that TCF7L1 upregulation by ONX-0914 is reversed by DON. (**F**) Co-immunoprecipitation (Co-IP) using anti-HA antibody in HA-tagged TCF7L1-overexpressing cells reveals O-GlcNAcylation of TCF7L1. (**G**) sWGA pull-down confirms that TCF7L1 is O-GlcNAc modified; modification is enhanced by PUGNAc and suppressed by OSMI-1. (**H**,**I**) Co-IP assays performed with anti-HA (**H**) or anti-Flag (**I**) in HA-TCF7L1/Flag-OGT co-transfected cells, demonstrating their interaction. (**J**) Co-IP shows native TCF7L1 also interacts with endogenous OGT in LNCaP cells. (**K**) Co-IP analysis shows ONX-0914 increases TCF7L1 O-GlcNAcylation. (**L**) sWGA pull-down further validates that ONX-0914 promotes O-GlcNAcylation of TCF7L1 in LNCaP cells. (**M**) Cell colony images showing reduced colonies density and proliferation in the OE TCF7L1 group compared to NC and siTCF7L1. (**N**) Quantification of cell colonies in each group. (**O**) CCK-8 assay demonstrates suppressed proliferation following TCF7L1 overexpression. (**P**,**Q**) Transwell assays and quantification indicate significantly reduced migration and invasion in the OE TCF7L1 group compared to controls. (**R**,**S**) qPCR (**R**) profiling of EMT-related transcripts and immunoblot (**S**) detection of EMT-associated proteins in NC, OE TCF7L1, and si TCF7L1 groups.

To identify transcription factors (TFs) that could link ONX-0914–induced metabolic changes to AR regulation, we performed an integrative analysis combining JASPAR-predicted AR-related TFs with differentially expressed genes from the TCGA-PRAD and the GSE46602 datasets. Thirteen TFs were common across the three sources (Fig. 4A). Validation by qPCR in prostate cancer cells revealed that TCF7L1, SPDEF and ETS2 were among the most consistently altered transcripts following ONX-0914 treatment (Fig. 4B). Given that TCF7L1 is functionally associated with Wnt signaling, and Wnt signaling is related to AR expression, we examined the effect of TCF7L1 on AR expression. qPCR and Western blot analyses demonstrated that TCF7L1 overexpression reduced AR mRNA and protein levels, whereas TCF7L1 knockdown increased AR expression (Fig. 4C,D). Furthermore, ONX-0914 treatment increased TCF7L1 protein levels in LNCaP cells, and this effect was reversed by DON, suggesting that ONX-0914 regulates the TCF7L1 expression via the HBP (Fig. 4E).

We next examined whether TCF7L1 undergoes O-GlcNAc modification. Co-immunoprecipitation (Co-IP) of HA-tagged TCF7L1 revealed robust O-GlcNAc signals on TCF7L1 (Fig. 4F), and sWGA pull-down assays confirmed that TCF7L1 is O-GlcNAcylated. This modification was enhanced by PUGNAc, an OGA inhibitor, and reduced by OSMI-1, an OGT inhibitor (Fig. 4G). Reciprocal Co-IP experiments demonstrated an interaction between TCF7L1 and OGT, supporting direct enzymatic modification (Fig. 4H–J). Consistently, ONX-0914 increased TCF7L1 O-GlcNAcylation, an effect that was blocked by OSMI-1 (Fig. 4K,L). Collectively, these results indicate that TCF7L1 is O-GlcNAcylated and that ONX-0914 promotes this modification.

We next investigated the functional role of TCF7L1 in HSPC cells. Overexpression of TCF7L1 significantly reduced colony formation compared with control or TCF7L1-silenced cells (Fig. 4M,N). CCK-8 assays confirmed that TCF7L1 overexpression markedly inhibited cell proliferation (Fig. 4O). Transwell assays demonstrated substantial decreases in both invasion and migration upon TCF7L1 overexpression (Fig. 4P,Q). Additionally, qPCR and Western blot analyses revealed that TCF7L1 overexpression downregulated mesenchymal markers (N-cadherin, vimentin) while upregulating the epithelial marker E-cadherin, consistent with the suppression of EMT (Fig. 4R,S). Collectively, these findings demonstrate that TCF7L1, a transcriptional repressor of AR, undergoes O-GlcNAcylation, which is enhanced by ONX-0914 via activation of the HBP. Functionally, TCF7L1 inhibits proliferation, invasion, migration, and EMT in HSPC cells. Furthermore, these results position TCF7L1 as a downstream effector of ONX-0914–induced HBP/O-GlcNAc activation and an upstream regulator of AR and EMT, supporting a model in which metabolic reprogramming converges on a transcriptional axis to restrain androgen-driven tumor progression.

### 3.5 O-GlcNAcylation Stabilizes TCF7L1 by Reducing Ubiquitin-Mediated Degradation, Enhancing Its Tumor-Suppressive Activity

**Figure 5:**
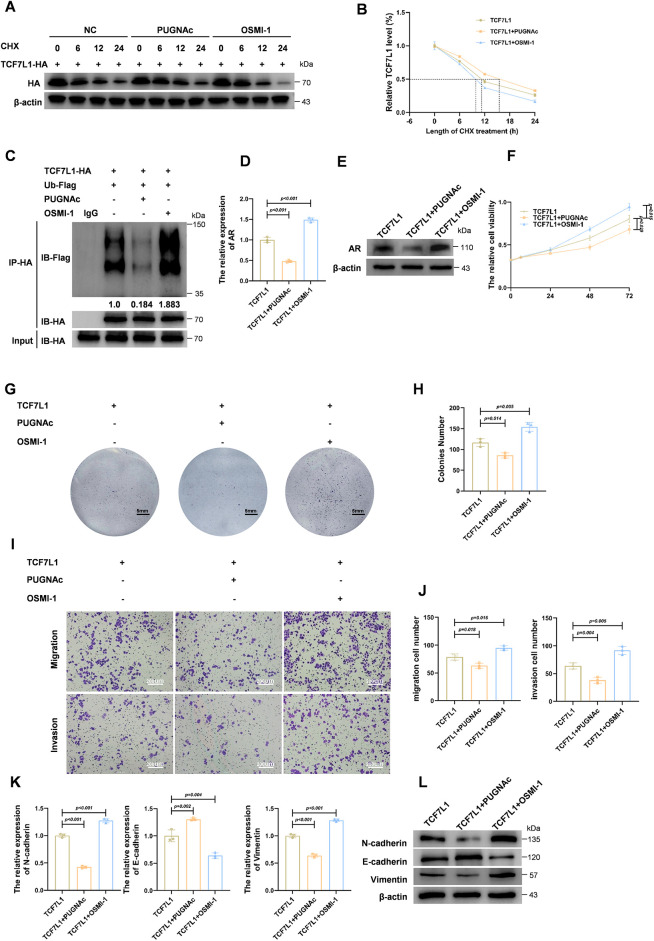
O-GlcNAcylation of TCF7L1 enhances its stability via reducing ubiquitin-mediated degradation, thereby augmenting tumor-suppressive activity. (**A**) Cycloheximide (CHX) chase assay showing the degradation kinetics of TCF7L1-HA protein in the presence of O-GlcNAcylation modulating agents. (**B**) Quantification of TCF7L1 protein half-life under the indicated treatments, showing stabilization by PUGNAc and destabilization by OSMI-1. (**C**) Co-immunoprecipitation of TCF7L1-HA followed by Flag immunoblotting in cells co-transfected with Ub-Flag reveals reduced ubiquitination of TCF7L1 upon PUGNAc treatment and increased ubiquitination upon OSMI-1 treatment. (**D**,**E**) qPCR (**D**) and Immunoblot analysis (**E**) showing that PUGNAc-mediated O-GlcNAcylation of TCF7L1 reduces AR expression, while OSMI-1 reverses this effect. (**F**) CCK-8 assay showing cell proliferation in TCF7L1-overexpressing cells with or without PUGNAc or OSMI-1. (**G**,**H**) Colony formation assay (**G**) and quantification (**H**) demonstrate that TCF7L1 + PUGNAc group forms significantly fewer colonies, whereas TCF7L1 + OSMI-1 group forms more and larger colonies compared to TCF7L1 alone. (**I**,**J**) Transwell assays and quantification indicate that O-GlcNAcylation of TCF7L1 reduces the invasive and migratory abilities of prostate cancer cells. (**K**,**L**) qPCR (**K**) and Immunoblot analysis (**L**) analyses of EMT markers in cells from TCF7L1, TCF7L1 + PUGNAc, and TCF7L1+ OSMI-1 groups.

To further investigate the role of TCF7L1 O-GlcNAcylation, we assessed its effect on protein stability. Cycloheximide (CHX) chase assays were performed in the presence of PUGNAc and OSMI-1. As illustrated in Fig. 5A, TCF7L1-HA degradation was markedly delayed following PUGNAc treatment, whereas OSMI-1 accelerated its degradation compared with the NC. Quantification of protein half-life (Fig. 5B) confirmed that increased O-GlcNAcylation stabilizes TCF7L1, whereas reduced O-GlcNAcylation promotes its degradation.

Afterward, we investigated the effect of O-GlcNAcylation on TCF7L1 ubiquitination using HA immunoprecipitation followed by Flag immunoblotting in cells co-expressing TCF7L1-HA and Ub-Flag. As shown in Fig. 5C, PUGNAc treatment significantly reduced TCF7L1 ubiquitination, whereas OSMI-1 treatment increased it, suggesting that O-GlcNAcylation protects TCF7L1 from proteasomal degradation by inhibiting its ubiquitination.

Functionally, we next assessed how O-GlcNAcylation of TCF7L1 affects AR expression. As shown by qPCR (Fig. 5D) and Western blot (Fig. 5E), PUGNAc treatment significantly decreased AR levels, whereas OSMI-1 reversed this effect. These findings indicate that TCF7L1 O-GlcNAcylation contributes to the downregulation of AR expression.

To further explore the functional impact of TCF7L1 O-GlcNAcylation on HSPC cellular behavior, we performed a series of cell-based assays using three experimental groups: TCF7L1 overexpression, TCF7L1 + PUGNAc, and TCF7L1 + OSMI-1. CCK-8 assays (Fig. 5F) demonstrated that inhibition of OGT by OSMI-1 significantly increased cell proliferation, whereas PUGNAc treatment further suppressed proliferation compared with TCF7L1 overexpression alone. Similarly, colony formation assays (Fig. 5G) showed that cells in the TCF7L1 + PUGNAc group formed significantly fewer colonies than those in the TCF7L1 group, while cells in the TCF7L1 + OSMI-1 group produced more and larger colonies. Quantitative analysis of colony numbers is illustrated in Fig. 5H.

Invasion and migration assays were then conducted (Fig. 5I,J). The fewest invading and migrating cells were observed in the TCF7L1 + PUGNAc group, while the highest numbers were seen in the TCF7L1 + OSMI-1 group. These results indicate that O-GlcNAcylation of TCF7L1 suppresses the pro-migratory and pro-invasive behavior of HSPC cells.

We also evaluated EMT marker expression using qPCR and Western blot. As shown in Fig. 5K,L, levels of N-cadherin and vimentin were further reduced in the TCF7L1 + PUGNAc group, whereas they were increased in the TCF7L1 + OSMI-1 group compared with TCF7L1 overexpression alone. Conversely, E-cadherin expression exhibited the opposite pattern. These results support that O-GlcNAcylation of TCF7L1 suppresses EMT progression.

Collectively, these findings demonstrate that O-GlcNAcylation stabilizes TCF7L1 by reducing ubiquitination-mediated degradation. Furthermore, TCF7L1 O-GlcNAcylation suppresses prostate cancer cell proliferation, invasion, migration, and EMT by downregulating AR expression.

### 3.6 O-GlcNAcylation of TCF7L1 Suppresses Prostate Cancer Growth In Vivo

**Figure 6:**
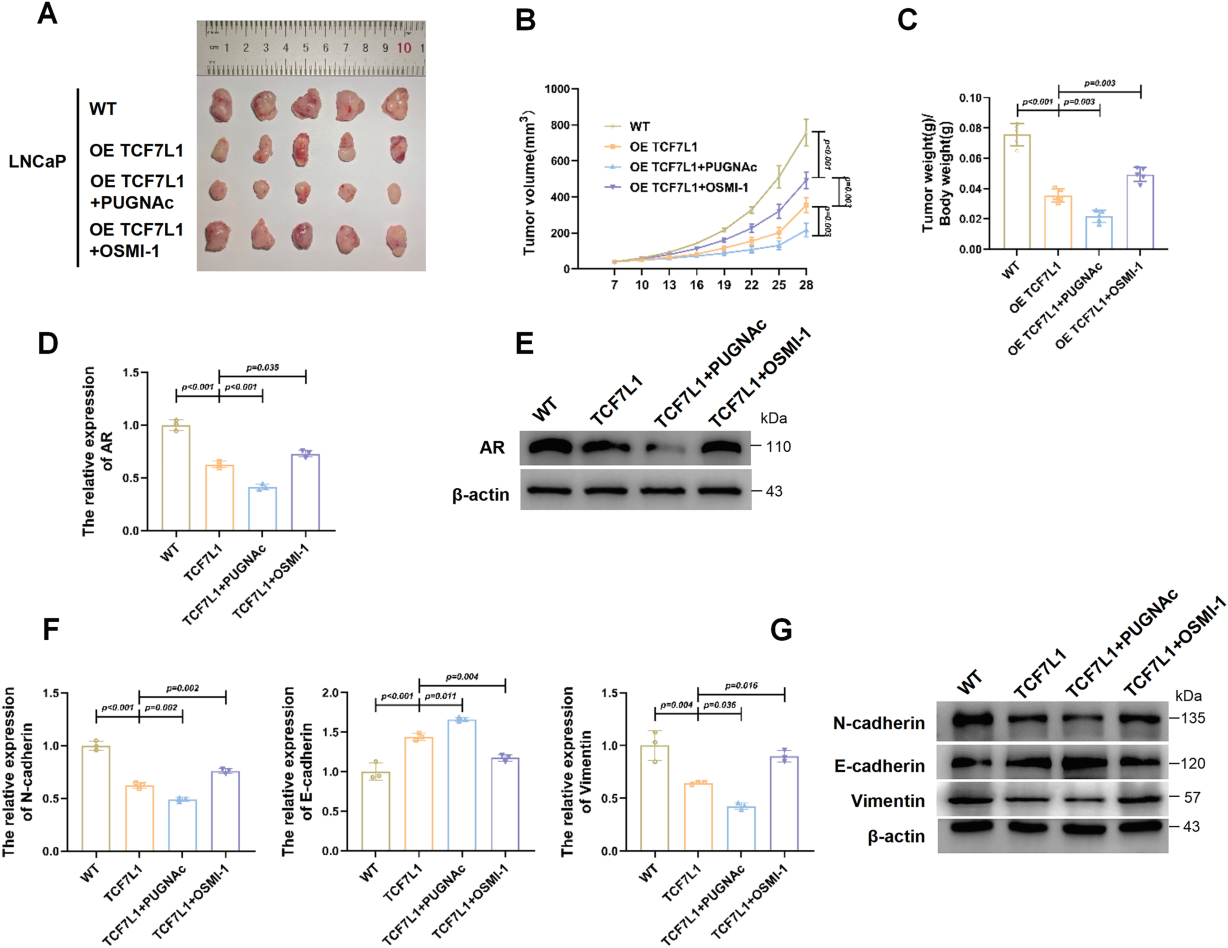
O-GlcNAcylation of TCF7L1 suppresses prostate cancer growth *in vivo*. (**A**) Representative images of xenograft tumors excised from nude mice. (**B**) Tumor volume measurements over time in each group. (**C**) Final tumor weights at the endpoint of the study. (**D**) qPCR analysis of AR mRNA expression in xenograft tumors. AR expression was highest in WT, followed by OETCF7L1 + OSMI-1, then OETCF7L1, and lowest in OETCF7L1 + PUGNAc. (**E**) Immunoblot validation of AR protein expression in tumor tissues from each group, confirming the qPCR trends. (**F**,**G**) qPCR analysis (**F**) and Immunoblot analysis (**G**) of EMT markers, demonstrating consistent EMT reversal in the OETCF7L1 + PUGNAc group and partial reversal in the OETCF7L1 + OSMI-1 group.

To further assess the functional significance of TCF7L1 and its O-GlcNAcylation *in vivo*, we established a xenograft model by implanting either parental LNCaP cells (WT) or TCF7L1-overexpressing stable LNCaP cells into mice, followed by intraperitoneal administration of OSMI-1 or PUGNAc. As shown in Fig. 6A, tumors were largest in the WT group, followed by the OETCF7L1 + OSMI-1 group, then the OETCF7L1 group, with the smallest tumors observed in the OETCF7L1 + PUGNAc group. Tumor volumetric and weight measurements (Fig. 6B,C) confirmed these findings, demonstrating a pronounced reduction in tumor growth in the OETCF7L1 + PUGNAc group compared with the other groups.

qPCR (Fig. 6D) and Western blot analyses (Fig. 6E) were performed to assess AR expression in tumor tissues. Both assays confirmed that AR levels were highest in WT tumors, moderately decreased in the OETCF7L1 + OSMI-1 group, further reduced in the OETCF7L1 group, and lowest in the OETCF7L1 + PUGNAc group.

To further evaluate the impact of TCF7L1 and its O-GlcNAcylation on EMT, the expression of EMT markers was analyzed. qPCR results (Fig. 6F) and Western blot analysis (Fig. 6G) revealed significant downregulation of mesenchymal markers (N-cadherin, vimentin) and pronounced upregulation of epithelial marker E-cadherin in the OETCF7L1 + PUGNAc group compared with other groups.

Collectively, these *in vivo* results demonstrate that TCF7L1 undergoes O-GlcNAcylation, which suppresses AR expression and consequently inhibits prostate cancer growth and EMT.

## 4 Discussion

Activation and dysregulation of AR signaling represent central drivers in the progression of prostate cancer, particularly during the transition from HSPC to CRPC [28]. In this study, we demonstrate that ONX-0914, a selective LMP7 inhibitor, exerts AR-suppressive and anti-proliferative effects in HSPC models. Originally developed to specifically target the immunoproteasome’s β5i/LMP7 subunit with high specificity, ONX-0914 has been shown to mediate antitumor activity in CRPC, mainly through immunomodulatory mechanisms [11]. Notably, previous studies have shown that genetic deletion of LMP7 in CRPC models exerts only limited effects on tumor growth, whereas pharmacological inhibition with ONX-0914 produces more pronounced antitumor activity. This discrepancy suggests that the antitumor effects of ONX-0914 may extend beyond direct LMP7 inhibition and involve additional context-dependent mechanisms. Therefore, the antiproliferative effect of ONX-0914 in HSPC seems not to be solely dependent on classical LMP7 inhibition. This observation indicates that ONX-0914 may act via an alternative mechanism in HSPC.

As a nutrient-regulated protein modification, O-GlcNAcylation involves the covalent addition of a single N-acetylglucosamine moiety to serine or threonine residues of nuclear and cytoplasmic proteins, catalyzed by O-GlcNAc transferase [29]. This process depends on UDP-GlcNAc, the end product of HBP, which reflects cellular glucose and glutamine availability [30]. While O-GlcNAcylation has been widely reported to promote oncogenesis [31], our data reveal a context-dependent role of O-GlcNAcylation. We show that enhanced O-GlcNAcylation strengthens the regulatory activity of TCF7L1, an AR-associated transcriptional repressor, leading to AR downregulation and tumor-suppressive effects. These findings challenge the notion that O-GlcNAcylation universally drives tumorigenesis. Indeed, evidence from multiple studies indicates that the functional consequences of O-GlcNAcylation are highly context-dependent, varying with the target protein and the surrounding cellular environment [32]. O-GlcNAcylation can modulate the activity of specific TFs in ways that either promote or restrain tumor progression, influenced by cellular conditions and cofactor interactions [33–35]. In the context of ONX-0914 treatment in HSPC cells, our results demonstrate that enhanced O-GlcNAcylation is associated with marked suppression of cell growth, highlighting the importance of substrate specificity in determining the functional outcome of this modification. Consistent with these observations, previous work has shown that the HBP activity is substantially reduced in CRPC cells, contributing to tumor progression via altered signaling pathways. Notably, supplementation with UDP-GlcNAc, the primary downstream metabolite of HBP, significantly decreased the viability and number of CRPC-like cells, suggesting that restoring HBP flux and increasing O-GlcNAcylation can exert tumor-suppressive effects in specific prostate cancer contexts [36].

Exposure of HSPC cells to ONX-0914 was associated with enhanced UDP-GlcNAc availability and a global increase in protein O-GlcNAcylation, which enabled the identification of TCF7L1 as a novel substrate of this modification. TCF7L1, a transcriptional regulator modulated by Wnt signaling [37], has been implicated in promoting tumorigenesis across multiple contexts [38,39]. In prostate cancer, recent studies suggest a contributory role for TCF7L1 in tumor progression. For example, ADT-induced WNT4 secretion has been reported to activate TCF7L1, driving IL-8/CXCR2 signaling and promoting neuroendocrine differentiation, with this induction related to deregulated AR signaling [40]. Conversely, TCF7L1 can also exhibit tumor-suppressive functions: in liver cancer and Ewing sarcoma, TCF7L1 acts as a critical metastasis suppressor, and its downregulation is associated with enhanced metastatic potential and poor prognosis [26,41]. In addition, studies in cervical and breast cancers have shown that specific mutations or downregulation of TCF7L1 are associated with a reduced risk of tumor development, further supporting its potential tumor-suppressive role [42,43]. Our data demonstrate that ONX-0914 treatment increases TCF7L1 expression in HSPC cells through activation of the HBP and enhanced O-GlcNAcylation. O-GlcNAcylation stabilizes TCF7L1 by inhibiting its ubiquitination-mediated degradation. Functionally, TCF7L1 stabilized via O-GlcNAcylation markedly reduces AR expression and suppresses EMT both *in vitro* and *in vivo*, resulting in tumor growth inhibition. Thus, TCF7L1 acts as a key mediator linking ONX-0914 to AR downregulation. Interestingly, whereas previous studies have associated TCF7L1 with neuroendocrine aggressiveness, our findings reveal a contrasting role: in the HSPC context, TCF7L1 functions as a tumor suppressor by restraining AR activity, highlighting the context-dependent plasticity of Wnt/TCF signaling in prostate cancer. Notably, hormone-sensitive and castration-resistant prostate cancer display well-established differences in metabolic states, immunoproteasome composition, and transcriptional programs. In CRPC, Th17-driven inflammation strongly upregulates immunoproteasome subunits such as LMP7, and ONX-0914 suppresses CRPC progression mainly through LMP7-dependent mechanisms [11]. In addition, key enzymes of the hexosamine biosynthesis pathway are markedly downregulated in CRPC, and supplementation with UDP-GlcNAc reduces proliferation and enhances sensitivity to enzalutamide, consistent with reduced HBP flux in CRPC [36]. In contrast, our HSPC models exhibit relatively low LMP7 expression, yet ONX-0914 remains highly effective and prominently activates the HBP/O-GlcNAc axis, indicating that metabolic–epigenetic regulation, rather than classical LMP7 inhibition, drives its antitumor effects in HSPC. Future studies will need to systematically assess TCF7L1 baseline expression, metabolic characteristics, and proteasome composition to fully clarify these context-dependent mechanisms.

Several limitations of this study should be acknowledged. Evidence for HBP activation was based on increased GFAT1 expression and increased global and TCF7L1-specific O-GlcNAcylation, rather than direct measurements of metabolic flux. While these findings suggest enhanced HBP activity, future metabolomic and enzymatic studies are needed to precisely define the metabolic impact of ONX-0914. Furthermore, the conclusion that ONX-0914 exerts effects not solely dependent on LMP7 in HSPC is primarily based on shRNA-mediated knockdown approaches. Although three independent shRNAs produced consistent results and knockdown efficiency was validated at both the mRNA and protein levels, additional genetic and biochemical approaches will be valuable to further substantiate this mechanism. The upstream mechanisms underlying GFAT1 induction were not directly investigated in the present study, and whether ONX-0914 regulates GFAT1 through direct transcriptional control or via broader metabolic or stress-associated responses remains to be determined. In addition, the current analyses were primarily conducted in a representative HSPC cell line. Validation in additional HSPC models, including alternative cell lines, organoids, or primary patient-derived tissues, will be important to assess the generalizability of the proposed HBP/O-GlcNAc/TCF7L1–AR axis. Finally, although LNCaP xenografts are widely used as a representative hormone-sensitive prostate cancer model, future studies incorporating direct biochemical assessments of androgen responsiveness, as well as genetically engineered models or clinical specimens, will be valuable for strengthening translational relevance.

Despite these limitations, our findings reveal a previously unrecognized metabolic–epigenetic mechanism by which ONX-0914 suppresses androgen-dependent prostate cancer. Future studies incorporating more direct assessments of immunoproteasome function, together with additional genetic and metabolic analyses, will be valuable for further refining the mechanistic framework proposed here. *In vivo* pharmacodynamic and toxicity assessments of ONX-0914 will further clarify its therapeutic potential in early-stage prostate cancer. These future investigations will be critical for defining the translational potential of targeting the HBP/O-GlcNAc/TCF7L1–AR axis in androgen-responsive prostate cancer.

## 5 Conclusion

In summary, this study uncovers a previously unrecognized metabolic–epigenetic mechanism by which ONX-0914 suppresses HSPC. By enhancing HBP-driven O-GlcNAcylation and stabilizing the transcriptional repressor TCF7L1, ONX-0914 reduces AR expression and inhibits malignant progression not solely dependent on classical LMP7-mediated immunoproteasome inhibition. Collectively, these results extend existing immunoproteasome-focused paradigms by revealing a metabolic–transcriptional mechanism that operates specifically in hormone-sensitive disease contexts. These findings highlight a context-dependent mode of action for immunoproteasome inhibitors and identify the HBP/O-GlcNAc/TCF7L1–AR axis as a potential therapeutic target in androgen-responsive disease. Future studies incorporating direct metabolic flux analyses, genetic perturbation of pathway components, and *in vivo* pharmacodynamic profiling will be crucial to assess the translational potential of targeting this regulatory network.

## Supplementary Materials





## Data Availability

The data that support the findings of this study are available from the Corresponding Authors upon reasonable request.

## Supplementary Materials

The supplementary material is available online at https://www.techscience.com/doi/10.32604/or.2026.073156/s1.

## Abbreviations

ADT: Androgen deprivation therapy
AR: Androgen receptor
CCK-8: Cell Counting Kit-8
CHX: Cycloheximide
Co-IP: Co-immunoprecipitation
CRPC: Castration-resistant prostate cancer
cDNA: Complementary DNA
DON: 6-Diazo-5-oxo-L-norleucine (GFAT1 inhibitor)
EMT: Epithelial–mesenchymal transition
ETS2: ETS proto-oncogene 2
GFAT1: Glutamine fructose-6-phosphate amidotransferase 1
GlcNAc: N-Acetylglucosamine
HA: Hemagglutinin
HBP: Hexosamine biosynthetic pathway
HSPC: Hormone-sensitive prostate cancer
LMP7: Low molecular mass polypeptide 7
MHC: Major histocompatibility complex
mRNA: Messenger RNA
O-GlcNAc: O-linked β-N-acetylglucosamine
O-GlcNAcylation: O-linked β-N-acetylglucosamine modification
OGA: O-GlcNAcase
OGT: O-GlcNAc transferase
OSMI-1: OGT inhibitor
PUGNAc: O-(2-acetamido-2-deoxy-D-glucopyranosylidene) amino-N-phenylcarbamate
qPCR: Quantitative PCR
siRNA: Small interfering RNA
SPDEF: SAM pointed domain containing ETS transcription factor
sWGA: Succinylated wheat germ agglutinin
TCF/LEF: T-cell factor/lymphoid enhancer factor
TCF7L1: Transcription factor 7-like 1
TFs: Transcription factors
