# Supplementary material for: ONX-0914 Suppresses Hormone-Sensitive Prostate Cancer by Promoting O-GlcNAcylation-Mediated Stabilization of TCF7L1
Source: Oncol Res. 2026 Mar 23;34(4):31. doi: 10.32604/or.2026.073156 (PMC13040289; doi:10.32604/or.2026.073156)
Supplement: Supplementary file 1 [file OncolRes-34-73156-s001.docx]

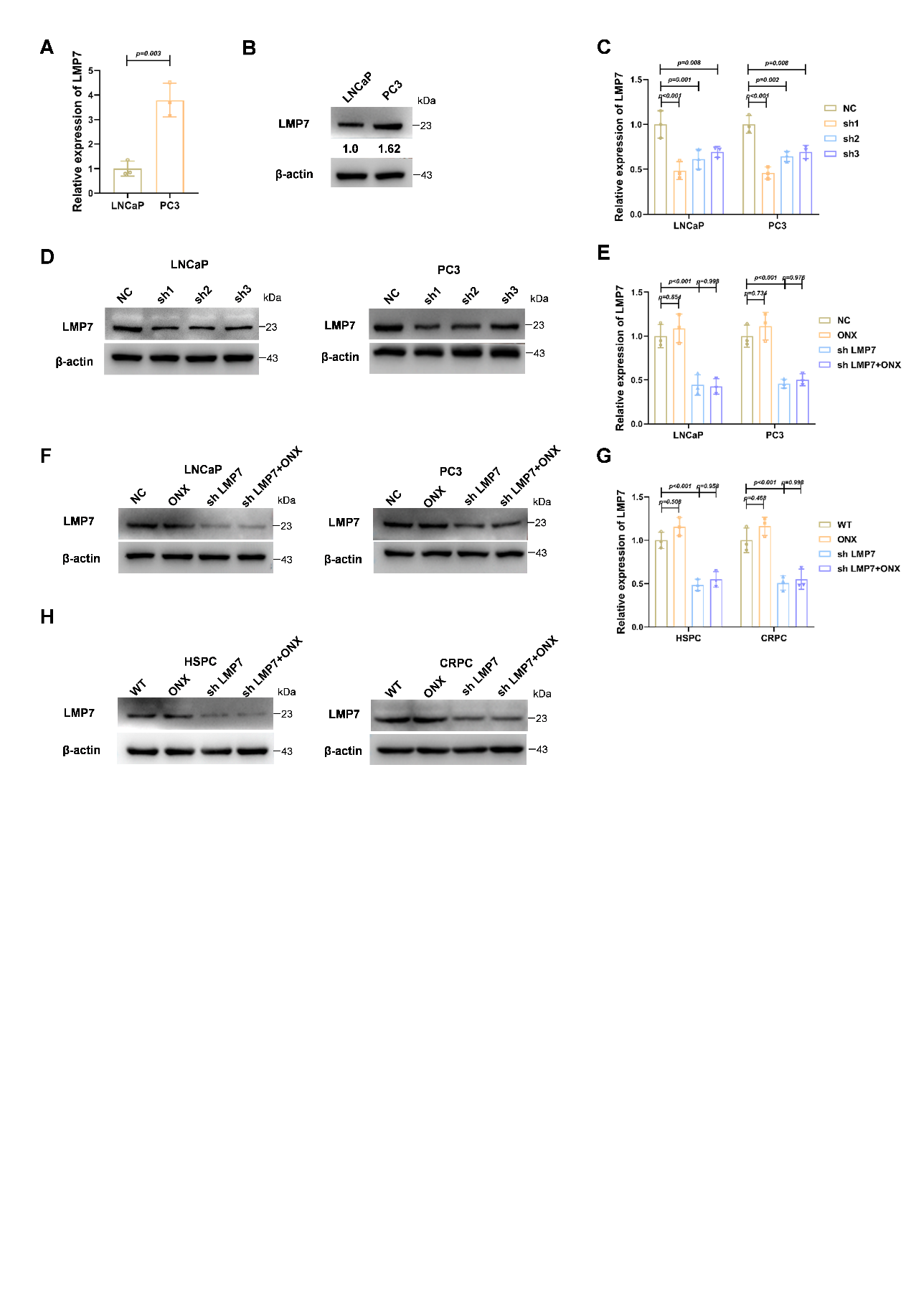


**Supplementary Figure S1. Low molecular mass polypeptide 7 (LMP7) expression and knockdown validation in prostate cancer cell lines and xenograft models.**
**(A)** Reverse transcription quantitative polymerase chain reaction (RT-qPCR) analysis of basal LMP7 mRNA expression in LNCaP and PC3 cells. **(B)** Immunoblot detection of LMP7 protein levels in LNCaP and PC3 cells. **(C)** RT-qPCR validation of LMP7 knockdown efficiency using three independent shRNAs (sh1–sh3) in LNCaP and PC3 cells. **(D)** Immunoblot validation of LMP7 knockdown in LNCaP and PC3 cells under the conditions shown in (C). **(E)** RT-qPCR analysis of LMP7 mRNA levels in LNCaP and PC3 cells treated with ONX-0914 (ONX) and/or shRNA targeting LMP7 (shLMP7), as indicated. **(F)** Immunoblot analysis of LMP7 protein in LNCaP and PC3 cells under the conditions described in (E). **(G)** RT-qPCR analysis of LMP7 expression in hormone-sensitive prostate cancer (HSPC) and castration-resistant prostate cancer (CRPC) xenograft tumors under the indicated treatments (wild type (WT), ONX, shLMP7, and shLMP7+ONX). **(H)** Immunoblot analysis of LMP7 protein in xenograft tumors.
