# Supplementary material for: ONX-0914 Suppresses Hormone-Sensitive Prostate Cancer by Promoting O-GlcNAcylation-Mediated Stabilization of TCF7L1
Source: Oncol Res. 2026 Mar 23;34(4):31. doi: 10.32604/or.2026.073156 (PMC13040289; doi:10.32604/or.2026.073156)
Supplement: Supplementary file 2 [file OncolRes-34-73156-s002.docx]

**Supplementary Table S1. Key Drugs**

| **Reagent** | **Catalog number** | **Supplier** | **City, State/Province, Country** |
| --- | --- | --- | --- |
| ONX-0914 | S7172 | Selleck Chemicals | Houston, TX, USA |
| Dimethyl sulfoxide (DMSO) | BL165A | Biosharp | Hefei, Anhui, China |
| DON | D2141 | Sigma-Aldrich | St. Louis, MO, USA |
| pentobarbital sodium | P3761 | Sigma-Aldrich | St. Louis, MO, USA |
| OSMI-1 | S9835 | Selleck Chemicals | Houston, TX, USA |
| PUGNAc | A7229 | Sigma-Aldrich | St. Louis, MO, USA |

**Supplementary Table S2.Cell lines and cell culture reagents**

| **Cell lines/ Reagent** | **Catalog number** | **Supplier** | **City, State/Province, Country** |
| --- | --- | --- | --- |
| PC3 | TCHu158 | Cell Bank, Chinese Academy of Sciences | Shanghai, China |
| LNCaP | TCHu173 | Cell Bank, Chinese Academy of Sciences | Shanghai, China |
| HEK293T | GNHu17 | Cell Bank, Chinese Academy of Sciences | Shanghai, China |
| RPMI-1640 medium | 11875-093 | Gibco, Thermo Fisher Scientific | Waltham, MA, USA |
| DMEM | 11965-092 | Gibco, Thermo Fisher Scientific | Waltham, MA, USA |
| Fetal bovine serum (FBS) | 164210 | Procell | Wuhan, Hubei, China |
| Penicillin–Streptomycin | C0222 | Beyotime Biotechnology | Shanghai, China |

**Supplementary Table S3. Primer, siRNA and shRNA sequences**

| **Item** | **Sequence (5’-3’)** |
| --- | --- |
| β-actin | Forward CCTTCCTGGGCATGGAGTC  Reverse TGATCTTCATTGTGCTGGGTG |
| N-cadherin | Forward TCAGGCGTCTGTAGAGGCTT  Reverse ATGCACATCCTTCGATAAGACTG |
| E-cadherin | Forward CGAGAGCTACACGTTCACGG  Reverse GGGTGTCGAGGGAAAAATAGG |
| Vimentin | Forward GACGCCATCAACACCGAGTT  Reverse CTTTGTCGTTGGTTAGCTGGT |
| GFAT1 | Forward AACTACCATGTTCCTCGAACGA  Reverse CTCCATCAAATCCCACACCAG |
| AR | Forward CCAGGGACCATGTTTTGCC  Reverse CGAAGACGACAAGATGGACAA |
| TCF7L1 | Forward TCGTCCCTGGTCAACGAGT  Reverse ACTTCGGCGAAATAGTCCCG |
| SPDEF | Forward CAGTGCCCGGTCATTGACA  Reverse CAGCCGGTATTGGTGCTCT |
| ETS2 | Forward CCCCTGTGGCTAACAGTTACA  Reverse AGGTAGCTTTTAAGGCTTGACTC |
| siRNA-AR | AAGAAGGCCAGUUGUAUGGAC |
| siRNA-TCF7L1 | AUCCGAGCUGUCACCGUAUUA |
| shNC | TTCTCCGAACGTGTCACGT |
| shLMP7-1 | CCACGTTAAGTCCAAGGAGAA |
| shLMP7-2 | CCTCTCTATGGGCAGTATGAT |
| shLMP7-3 | GTTGGGTGAAAGTAGAAAGTA |

**Supplementary Table S4. Reagents Used for Transfection and Lentiviral Transduction**

| **Plasmid / Vector** | **Catalog number** | **Supplier** | **City, State/Province, Country** |
| --- | --- | --- | --- |
| pcDNA3.1 | V79020 | Invitrogen | Waltham, MA, USA |
| psPAX2 | 12260 | Addgene | Watertown, MA, USA |
| pMD2.G | 12259 | Addgene | Watertown, MA, USA |
| Lipofectamine 3000 | L3000015 | Thermo Fisher Scientific | Waltham, MA, USA |
| Puromycin | HB-PU-500 | Hanbio | Shanghai, China |
| Polybrene | H9268 | Sigma-Aldrich | St. Louis, MO, USA |

**Supplementary Table S5. RNA extraction and RT–qPCR reagents**

| **Reagent** | **Catalog number** | **Supplier** | **City, State/Province, Country** |
| --- | --- | --- | --- |
| TRIzol reagent | RK30129 | ABclonal | Wuhan, Hubei, China |
| ABScript Neo RT Master Mix for qPCR | RK20433 | ABclonal | Wuhan, Hubei, China |
| SYBR Green Fast qPCR Mix | RK21203 | ABclonal | Wuhan, Hubei, China |

**Supplementary Table S6. Western blotting reagents**

| **Reagent** | **Catalog number** | **Supplier** | **City, State/Province, Country** |
| --- | --- | --- | --- |
| RIPA lysis buffer | P0013B | Beyotime Biotechnology | Shanghai, China |
| Protease inhibitor cocktail | P1005 | Beyotime Biotechnology | Shanghai, China |
| Color Prestained Protein Ladder, 15–150 kDa | WJ101 | EpiZyme | Shanghai, China |
| GFAT1 | 14132-1-AP | Proteintech | Chicago, IL, USA |
| AR | 22089-1-AP | Proteintech | Chicago, IL, USA |
| TCF7L1 | 14519-1-AP | Proteintech | Chicago, IL, USA |
| Flag | 20543-1-AP | Proteintech | Chicago, IL, USA |
| Anti-HA (hemagglutinin) | 51064-2-AP | Proteintech | Chicago, IL, USA |
| E-cadherin | 20874-1-AP | Proteintech | Chicago, IL, USA |
| N-cadherin | 22018-1-AP | Proteintech | Chicago, IL, USA |
| Vimentin | 10366-1-AP | Proteintech | Chicago, IL, USA |
| β-actin | 66009-1-Ig | Proteintech | Chicago, IL, USA |
| O-GlcNAc | PTM-952 | Jingjie PTM BioLab | Hangzhou, Zhejiang, China |
| LMP7 | ET7107-36 | Huabio | Hangzhou, Zhejiang, China |
| HRP Conjugated AffiniPure Goat Anti-Mouse IgG (H+L) | BA1050 | Boster Biological Technology | Pleasanton, CA, USA |
| HRP Conjugated AffiniPure Goat Anti-Rabbit IgG (H+L) | BA1054 | Boster Biological Technology | Pleasanton, CA, USA |

**Supplementary Table S7. sWGA pull-down and CHX chase reagents**

| **Reagent** | **Catalog number** | **Supplier** | **City, State/Province, Country** |
| --- | --- | --- | --- |
| PNGase F | P0704S | New England Biolabs | Ipswich, MA, USA |
| Succinylated Wheat Germ Agglutinin (WGA), Biotinylated | B-1025S-5 | Vector Laboratories | Newark, CA, USA |
| Cycloheximide (CHX) | C7698 | Sigma-Aldrich | St. Louis, MO, USA |
| OSMI-1 | S9835 | Selleck Chemicals | Houston, TX, USA |
| PUGNAc | A7229 | Sigma-Aldrich | St. Louis, MO, USA |
| GlcNAc | A8625 | Sigma-Aldrich | St. Louis, MO, USA |

**Supplementary Table S8. Co-immunoprecipitation and ubiquitination assay reagents**

| **Reagent** | **Catalog number** | **Supplier** | **City, State/Province, Country** |
| --- | --- | --- | --- |
| OSMI-1 | S9835 | Selleck Chemicals | Houston, TX, USA |
| PUGNAc | A7229 | Sigma-Aldrich | St. Louis, MO, USA |
| N-ethylmaleimide（NEM） | E3876 | Sigma-Aldrich | St. Louis, MO, USA |
| Protein A/G magnetic beads | 88803 | Thermo Fisher Scientific | Waltham, MA, USA |
| IP lysis buffer | P0013 | Beyotime Biotechnology | Shanghai, China |

**Supplementary Table S9. CCK-8 and Transwell**

| **Reagent** | **Catalog number** | **Supplier** | **City, Province/State, Country** |
| --- | --- | --- | --- |
| CCK-8 | CK04 | Dojindo Laboratories | Mashiki-machi, Kumamoto, Japan |
| Transwell | 3422 | Corning | Corning, NY, USA |
| Matrigel | 356234 | Corning | Corning, NY, USA |
| Crystal violet | C0121 | Beyotime Biotechnology | Shanghai, China |
| Paraformaldehyde | P0099 | Beyotime Biotechnology | Shanghai, China |
